# On Styptic Applications; Communicated to Dr. Bradley by Mr Edward Thornhill Luscombe, of the Second Dragoons

**Published:** 1802-09-01

**Authors:** Edward Thornhill Luscombe


### *On Styptic Applications; communicated to Dr. Bradley by* MR EDWARD THORNHILL LUSCOMBE, *of the Second Dragoons.*

DURING the time I did duty at the General Military Hofpital at Gofport, (to which place I went immediately after I ceafed to be a pupil of your's, in Auguft, 1801,) I had a confiderable number of men from Egypt and the Weft Indies in my charge, under the fuperintendance of Dr. Rogerfon, and the Surgeons to the forces. Amongft my patients were many with wounds, principally gun-fhot, in a very bad ftate indeed;

 moft moſt of their ſtumps were in a ſloughing or gangrenous ſtate, and hæmorrhagies from them took place almoſt hourly. In ſeveral caſes the preſſure which could be applied, together with the ſtyptics in common uſe, were inſufficient to reſtrain the flow of blood. In theſe men the hæmorrhage was effectually ſtopped by a ſolution of antimonium tartariſatum, in the proportion of four grains to an ounce of water, applied to the wounds by means of lint completely wetted in it. The parts were in ſuch a ſtate that the application of a ligature was impracticable.

The action of the antim. tart. I muſt leave to be explained by older and more able phyſiologiſts, not feeling myſelf at all equal to the taſk: But being convinced that it preſerved the life of more than one man, I beg leave to requeſt that you will give the naked fact a place in your univerſally read Journal, hoping that in other hands it will prove equally ſucceſsful.

